# Antidepressant-like effects of methanol extract of *Hibiscus tiliaceus* flowers in mice

**DOI:** 10.1186/1472-6882-12-41

**Published:** 2012-04-12

**Authors:** Cláudia Vanzella, Paula Bianchetti, Sabrina Sbaraini, Samanta Inês Vanzin, Maria Inês Soares Melecchi, Elina Bastos Caramão, Ionara Rodrigues Siqueira

**Affiliations:** 1 Centro de Ciências Biológicas e da Saúde, Centro Universitário UNIVATES, Rua Avelino Tallini, 171, Bairro Universitário, CEP 95900-000, Lajeado, RS, Brazil; 2 Instituto de Química, Universidade Federal do Rio Grande do Sul, Av. Bento Gonçalves, 9500, Bairro Agronomia, 91501-970, Porto Alegre, RS, Brazil; 3 Departamento de Farmacologia, Universidade Federal do Rio Grande do Sul, Rua Sarmento Leite, 500 sala 202, 90050-170, Porto Alegre, RS, Brazil

## Abstract

**Background:**

*Hibiscus tiliaceus* L. (Malvaceae) is used in postpartum disorders. Our purpose was to examine the antidepressant, anxiolytic and sedative actions of the methanol extract of *H. tiliaceus* flowers using animal models.

**Methods:**

Adult male Swiss albino mice were treated with saline, standard drugs or methanol extract of H. tiliaceus and then subjected to behavioral tests. The forced swimming and tail suspension tests were used as predictive animal models of antidepressant activity, where the time of immobility was considered. The animals were submitted to the elevated plus-maze and ketamine-induced sleeping time to assess anxiolytic and sedative activities, respectively.

**Results:**

Methanol extract of *H. tiliaceus* significantly decreased the duration of immobility in both animal models of antidepressant activity, forced swimming and tail suspension tests. This extract did not potentiate the effect of ketamine-induced hypnosis, as determined by the time to onset and duration of sleeping time.

**Conclusion:**

Our results indicate an antidepressant-like profile of action for the extract of *Hibiscus tiliaceus* without sedative side effect.

## Background

*Hibiscus tiliaceus* L. (Malvaceae, beach hibiscus) is frequently found in coastal ecosystems and the tree is native to the shores of the Pacific and Indian oceans; today it is naturalized throughout the tropical and subtropical regions of the world, including southern Brazil. In addition to its use as an ornamental tree, *H. tiliaceus* has some medicinal uses. It has been reported that *H. tiliaceus* flowers possess properties that are useful against bronchitis, as well as in the treatment of fevers and coughs, ear infections and abscesses, postpartum disorders and skin diseases [1-6]. This species is consumed as a beverage (tea) in southern Brazil; however, its pharmacological effects and chemical composition are still poorly studied; some members of the genus *Hibiscus* produce a variety of bioactive compounds, such as lignanamides, naphthalenes, polyphenols, carotenoids, tocopherols, flavonoids, anthocyanins, phytosterols and long chain fatty esters [7].

Some studies have demonstrated that other species from this genus possess various biological effects. *H. sabdariffa* L. dried flowers have been used in folk medicines against hypertension, pyrexia and liver disorders. It has been demonstrated that anthocyanins from *H. sabdariffa* exhibited antihepatotoxic and antioxidative effects. These compounds were able to quench the free radicals DPPH and were effective in the model of tert-butyl hydroperoxide (t-BHP)-induced cytotoxicity in rat primary hepatocytes and hepatotoxicity in rats [8]. *H. sabdariffa* shows also antihypertensive and cardioprotective effects *in vivo*, what includes patients with moderate essential hypertension, where there are a reduction in systolic and diastolic pressure [9].

Recently, it was found that vitamin E and several phytosterols, such as stigmasterol, stigmastadienol and stigmastadienone, are present in the methanol extract of *H. tiliaceus*[10]*.* This extract showed antigenotoxic and antimutagenic effects against oxidative DNA damage induced by hydrogen peroxide (H_2_O_2_) and tert-butyl hydroperoxide in V79 cells; these cells are frequently used for studies on DNA damage and DNA repair [6,11]. Methanol extract of *Hibiscus tiliaceus* per se at concentrations ranging from 0.001 to 0.1 mg/mL was not cytotoxic, genotoxic or mutagenic. The treatment with non-cytotoxic concentrations increased cell survival after H_2_O_2_ and tert-butyl hydroperoxide exposure and prevented DNA damage. Besides, the methanol extract of *Hibiscus tiliaceus* prevented the increase in lipid peroxidation and decrease in GSH content [6,11].

As *H. tiliaceus* is used in postpartum disorders [5], it is important to consider the extent to which such conditions are present. Postpartum affective disorders occur following approximately 10% of obstetrical deliveries and can constitute a disabling illness with considerable impact on family and neonatal well-being [12,13]. Also, postpartum depression has a significant effect on the cognitive and emotional development of children [14]. Despite being a distinct subtype of major depressive disorder the underlying physiology of postpartum depression has received little attention. In addition, postpartum anxiety disorders are underemphasized and may be more common than depression [15]. Furthermore, there is evidence that the puerperium may exacerbate or induce new episodes of 'panic disorder’ [16].

Moreover, coumarins and coumarino-lignans from *H. syriacus* root bark have been found to inhibit the activity of monoamine oxidase [17], an enzyme which catalyzes the oxidative deamination of biogenic amines. Since this is the mechanism of action of one class of antidepressant drugs, and *H. tiliaceus* is popularly used in postpartum disorders, we hypothesized that *H. tiliaceus* might has antidepressant- and/or anxiolytic-like effect.

Therefore, the aim of this study was to examine the antidepressant-like, anxiolytic-like and sedative actions of the methanol extract of *H. tiliaceus* flowers using animal models. Putative antidepressant-like and anxiolytic-like properties of *H. tiliaceus* were studied in the forced swimming test, tail suspension test and elevated plus-maze test, while the sedative action was investigated by the ketamine-induced sleeping time.

## Methods

### Plant material

*H. tiliaceus* L. flowers were collected in the mangroves of Santa Catarina, Brazil, in December 2003 and January 2004. As it is a seasonal plant, it was not possible to perform another collection during the year. The plant was identified by Dr. B. Irgang (Instituto de Biociências, UFRGS, RS, Brazil). A dried voucher specimen (ICN: 113936) has been deposited in the Herbarium of UFRGS.

### Preparation of methanol extract

Fifteen grams of dried and ground flowers were continuously extracted for 48 h with methanol in a Soxhlet apparatus. The extract was filtered and concentrated in a rotary evaporator at 30–40°C to obtain semi-solid material. The viscous residue thus obtained was transferred to a vacuum desiccator over phosphorus pentoxide for 24 h to obtain a completely dry solid mass [18]. The extract was dissolved in distilled water for administration to the animals.

### Animals and treatment

Adult male Swiss albino mice (CF1 strain) aged 3 months were used, housed at a temperature of 22 ± 1°C under a light–dark cycle of 12 h and with access to food and water ad libitum. The NIH “Guide for the Care and Use of Laboratory Animals” (NIH publication No. 80–23, revised 1996) was followed in all experiments. All experimental protocols and care of animals were approved by the Local Ethical Committee (Centro Universitário UNIVATES). Saline, standard drugs (positive controls) and the methanol extract of *H. tiliaceus* (3, 10 and 30 mg/kg) were administered intraperitoneally (0.1 ml/10 g). Each experimental group consisted of at least 10 animals. Behavioral observations took place in soundproof rooms during the same period of the day to reduce the confounding influence of diurnal variation in spontaneous behavior. Each animal was tested only once.

### Forced swimming test

The method used was similar to that described by Porsolt and colleagues [19]. Animals were randomly divided into four groups. Thirty minutes after the i.p. treatment with the methanol extract of *H. tiliaceus* (3, 10 and 30 mg/kg) or nortriptyline HCl (Pamelor®, 2 mg/kg), a reference antidepressant drug, or saline, mice were individually placed in an open cylindrical container (35 cm height × 24 cm diameter) containing 13.5 cm of water at 22–25°C for 6 min. The duration of immobility was scored during the last 4 min of the 6-min test period. Mice were recorded as immobile when floating motionless or making only those movements necessary to keep the head above water. A decrease in the duration of immobility during the forced swimming test was taken as a measure of antidepressant activity.

### Tail suspension test

The total duration of immobility induced by tail suspension was measured according to the method of Steru and colleagues [20]. Mice were suspended 50 cm above the floor by adhesive tape placed approximately 1 cm from the tip of the tail. Immobility time was recorded during a 6-min test.

### Elevated plus-maze test (EPM)

This test has been widely validated to measure anxiety in rodents [21,22]. The plus-maze was a modification of that validated for mice by Lister [22] and comprised two open (30x5x25 cm) and two enclosed (30x5x25 cm) arms which extended from a common central platform (5x5 cm). The apparatus was made of wood, and was elevated to a height of 45 cm above floor level. To facilitate exploration, an edge (0.25 cm) was included around the perimeter of the open. Thirty minutes after the i.p. treatment with the methanol extract of *H. tiliaceus* (3, 10 and 30 mg/kg) or diazepam (0.5 mg/kg), a reference anxiolytic drug, or saline, each animal was placed at the center of the maze, facing one of the open arms. During the 5-min test period, the number of open and enclosed arms entries, as well as the time spent in open and enclosed arms, was recorded [22]. Entry into an arm was defined as the point when the animal placed all four paws onto the arm. After the test the maze was carefully cleaned with ethanol solution.

### Ketamine-induced sleeping time

The effect of plant extracts on ketamine-induced sleeping time was measured as described by Mimura and colleagues [23]. Thirty min after an i.p. injection of the methanol extract of *H. tiliaceus* (3, 10 and 30 mg/kg), standard drug (0.5 mg/kg) or vehicle, animals were injected with ketamine (100 mg/kg, i.p.). The interval between the administration of ketamine and the loss of the righting reflex was considered as the time to onset of sleep, while the time from the loss to regaining of the righting reflex was taken as the duration of sleep [24].

### Statistical analysis

The results were expressed as median (25th/75th of percentiles) or mean (± S.E.M.) values. Before statistical testing data were explored in order to assess their pattern of distribution. Kruskal-Wallis or ANOVA, followed by Dunn or Tukey Tests, respectively, were then employed considering the distribution of the data. Significance was assumed as *p* < 0.05.

## Results

**Figure 1:**
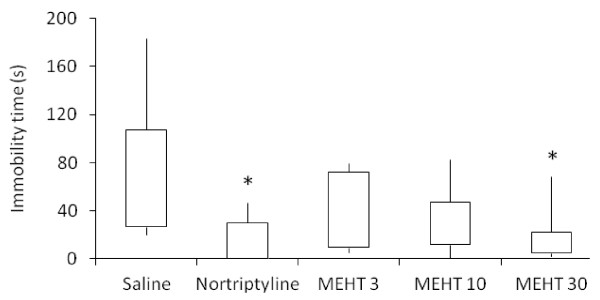
**Effect of*H. tiliaceus*on performance in the forced swimming test.** Effect of methanol extract of *H. tiliaceus* (MEHT; 3, 10 and 30 mg/kg) and nortriptyline (2 mg/kg) on performance in the forced swimming test (n = 10). The results were expressed as median, 25th percentile, 75th percentile, minimum and maximum values. *p < 0.05 compared to saline group (Kruskal-Wallis followed by Dunn test).

**Figure 2:**
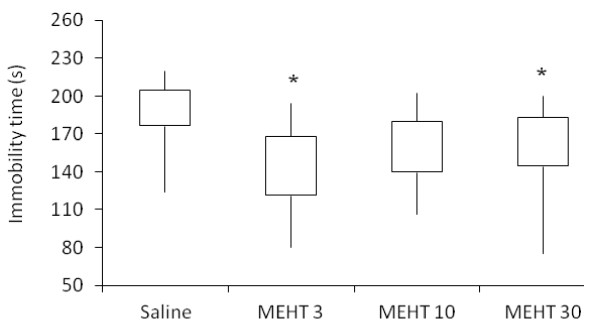
**Effect of*H. tiliaceus*on performance in the tail suspension test.** Effect of methanol extract of *H. tiliaceus* (MEHT) on performance in the tail suspension test (n = 10). The results were expressed as median, 25th percentile, 75th percentile, minimum and maximum values. *p < 0.05 compared to saline group (Kruskal-Wallis followed by Dunn test).

The methanol extract of *H. tiliaceus* showed antidepressant-like activity in predictive animal models, namely forced swimming and tail suspension tests. The extract (30 mg/kg) and nortriptyline significantly decreased the duration of immobility in the forced swimming test (Figure 1; Kruskal-Wallis test, H (4) = 14.230; *p* = 0.007). Dunn’s post hoc analysis demonstrated that both treatments significantly shortened the immobility time in comparison to the saline group (*p* < 0.05). Likewise, the extract reduced the duration of immobility in the tail suspension test (Figure 2; Kruskal-Wallis test, H (3) = 15.440; *p* = 0.001). Post hoc analysis demonstrated that the extract (3 and 30 mg/kg) significantly decreased the immobility time in comparison to the saline group (*p* < 0.05), although there are no significant differences among tested doses.

**Figure 3:**
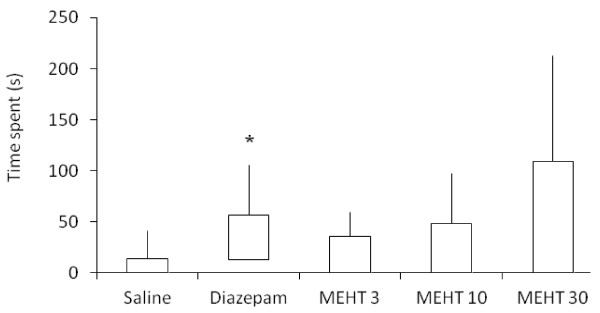
**Effect of*H. tiliaceus*on performance in the elevated plus-maze test.** Effect of methanol extract of *H. tiliaceus* (MEHT; 3, 10 and 30 mg/kg) and diazepam (0.5 mg/kg) on performance in the elevated plus-maze test (n = 10). The time spent in the open arms was expressed as median, 25th percentile, 75th percentile, minimum and maximum values. *p < 0.05 compared to saline group (Kruskal-Wallis followed by Dunn test).

As shown in Figure 3, in the elevated plus-maze test diazepam significantly increased the time spent in the open arms. Meanwhile, animals treated with the methanol extract of *H. tiliaceus* at 30 mg/kg showed a trend towards increased time spent in these arms, although this did not reach significance.

**Figure 4:**
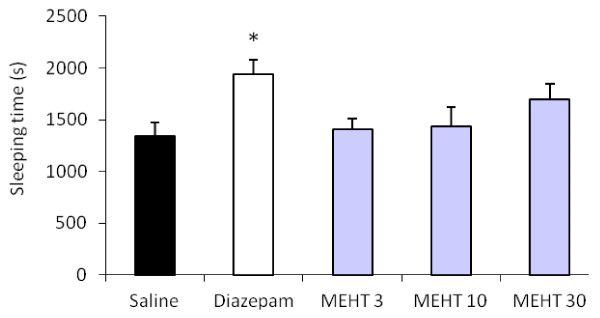
**Effect of*H. tiliaceus*on sleeping time induced by ketamine.** Effect of methanol extract of *H. tiliaceus* (MEHT; 3, 10 and 30 mg/kg) and diazepam (0.5 mg/kg) on sleeping time induced by ketamine (100 mg/kg, i.p.) (n = 10). The sleeping time was expressed as mean (± S.E.M.) values. *p < 0.05 compared to saline group (ANOVA followed by Tukey test).

The methanol extract of *H. tiliaceus* did not potentiate the effect of ketamine-induced hypnosis at the tested doses, as determined by the time to onset and duration of sleeping time, since the ketamine-induced loss of the righting reflex was unaltered by treatment (Figure 4). *H. tilliaceus* extract given alone did not show any sedative properties.

The soxhlet extraction with methanol showed a yield of 12.45 ± 0.23% (mass yield). This yield is very high for this kind of extraction and is in accord with the polarity of the solvent.

## Discussion

The present study provides behavioral evidence for the antidepressant-like activities of *H. tiliaceus*. The tail suspension and forced swimming tests are widely accepted behavioral models for the assessment of antidepressant activity [19,25]. The characteristic behavior evaluated in these tests, termed immobility, has been considered to reflect behavioral despair similar to that seen in human depression, and it is well known that antidepressant drugs are able to reduce the immobility time in rodents [19]. It is interesting to note that despite other works have used higher doses of plant extracts; in our study smaller doses were effective, what can indicate a high potency of this extract.

Interestingly, our data indicate an antidepressant-like profile of action for the extract of *Hibiscus tiliaceus* without sedative side effect, since the methanol extract of *H. tiliaceus* did not potentiate the effect of ketamine-induced hypnosis, moreover this extract given alone did not produce any sedation per se. In addition, this result may exclude pharmacokinetic interactions between methanol extract of *H. tiliaceus* and ketamine.

Administration of the methanol extract of *H. tiliaceus* showed a trend to increase the time spent in the open arms, suggesting a reduction in anxiety-like behavior, although this was not significantly different from the control group. However, this result cannot be disregarded, since many clinical effects appear only after chronic treatment. Different doses, treatment schedules, and additional anxiety-related models might be tested. Besides, although the effect of antidepressant treatments on performance in the elevated plus-maze is controversial, acute antidepressants have frequently demonstrated an anxiogenic-like profile in this paradigm [26-28]. Our extract did not induce any anxiety behavior. Taken together these evidences may suggest an uncommon and novel antidepressant/anxiolytic mechanism of action for compounds present in the methanol extract of *H. tiliaceus*.

It is interesting to note that *H. tiliaceus* has been traditionally used in postpartum disorders, whereas phytosterols, such as stigmasterol, stigmastadienol and stigmastadienone, found in the methanol extract of *H. tiliaceus*, might be useful in the treatment or prevention of postpartum depression related to withdrawal from chronic high levels of pregnancy-associated hormones, similar to obtained with estradiol administration [29]. Galea and collaborators [29] suggested that postpartum depression implies the withdrawal of chronically high levels of pregnancy-associated hormones (estradiol and progesterone), producing depression symptomatology in animal models that can be prevented by long term administration of high levels of estradiol through the postpartum period.

There is substantial evidence for the role of neuroactive steroids in the pathophysiology of major depression and related clinical conditions, such as premenstrual dysphoric disorder or postpartum depression, in addition to anxiety disorders. Furthermore, it has been suggested that neuroactive steroids may contribute to the therapeutic effects of antidepressants. Neuroactive steroids, such as pregnenolone sulfate and dehydroepiandrosterone sulfate decrease immobility time in the forced swimming procedure [30-32], suggesting an antidepressant-like profile. In addition, administration of progesterone was effective in the treatment of postpartum depression and premenstrual dysphoric disorder [33-35].

It has been suggested that a reduction in 3α-pregnane neuroactive steroids is related to the pathophysiology of major depression [36,37]. 3α-reduced neuroactive steroids (3α, 5α-tetrahydroprogesterone - THP; 3α, 5β-THP; 3α, 5α-tetrahydrodeoxycorticosterone) are potent allosteric modulators of GABA_A_-receptors [38,39]. Moreover, 3α, 5α-THP and 3α, 5β-THP are decreased in major depression, while there is an increase in 3β, 5α-THP (3β-hydroxy-5α pregnan-20-one; isopregnanolone) [36], which is an antagonist for GABA-agonistic steroids. In addition, antidepressant-treatment with fluoxetine counteracts this alteration in 3α-pregnane steroids [36,37,40,41]. Rupprecht and Zwanzger [39] suggested that selective serotonin reuptake inhibitors might be effective in the treatment of panic disorder by reducing the imbalance of endogenous neuroactive steroids during panic attacks. It is important to note that the neuroactive steroids 3α, 5α-THP and 3α, 5α-THDOC attenuated anxiety-related behavior without affecting spontaneous locomotor activity [42,43].

Taken together, we can suggest that phytosterols, such as stigmasterol, stigmastadienol and stigmastadienone, found in the methanol extract of *H. tiliaceus*, might be related to treatment or prevention of postpartum depression related to withdrawal from chronic high levels of pregnancy-associated hormones. Also, we can propose that these phytosterols might be effective in the treatment of depression and anxiety disorders by revert the imbalance of endogenous neuroactive steroids. Our finding can suggest an innovative mechanism of action.

Further support for the significance of our results is the finding that coumarins and coumarino-lignans of *H. syriacus* root bark have a mechanism of action as one class of antidepressant drugs, inhibiting the activity of monoamine oxidase [17], which catalyzes the oxidative deamination of the biogenic amines norepinephrine, serotonin and dopamine. The inhibition of monoamine oxidase activity might be involved to antidepressant-like activity of methanol extract of *H. tiliaceus*.

Recently, oxidative stress was linked with the pathophysiology of major depression, with significant correlations being found between the severity of depression and erythrocyte superoxide dismutase/lipoperoxidation levels [44]. Meanwhile, treatment with antidepressants reduces the oxidative stress related to depressive disorder [45,46]. Additionally, some species such as *Bacopa monneiraWithania somnifera* and *Asparagus racemosus*, all of which are reported to have antidepressant properties, also possess antioxidant activity [47-49]. Therefore, it is possible that the antioxidant activity of the methanol extract from *H. tiliaceus*[10,11] may contribute to its antidepressant-like effect.

## Conclusions

The methanol extract of *H. tiliaceus* possesses an antidepressant-like activity without sedative side effect. However, further neurochemical studies will be necessary to clarify its mechanism of action and to characterize the active principles.

## Competing interests

The authors declare that they have no competing interests.

## Author’s contributions

IRS performed the study and drafted the manuscript. CV, PB, SS and SIV carried out all behavioral tests in animals and performed the statistical analysis of data. MISM and EBC performed the preparation of methanol extract of *Hibiscus tiliaceus*. All authors read and approved the final manuscript.

## Pre-publication history

The pre-publication history for this paper can be accessed here:

http://www.biomedcentral.com/1472-6882/12/41/prepub
